# Clinical Outcomes Observed among Biopsy Proven Lupus Nephritis Patients Treated with Mycophenolate Mofetil as First-line Therapy

**DOI:** 10.7759/cureus.1907

**Published:** 2017-12-04

**Authors:** Homa Timlin, Laurence Magder, Michelle Petri

**Affiliations:** 1 Medicine, The Johns Hopkins University School of Medicine; 2 Epidemiology and Public Health, University of Maryland School of Medicine; 3 Rheumatology, The Johns Hopkins University School of Medicine

**Keywords:** proteinuria, urine protein creatinine ratio, esrd (end stage renal disease), mycophenolate mofetil, systemic lupus erythematosus, lupus nephritis, bms (bristol myers-squibb), alms (aspreva lupus management study), acr (american college of rheumatology), lunar (lupus nephritis assessment with rituximab)

## Abstract

Background and objective

The rate of end-stage renal disease from lupus nephritis has not declined, in spite of recent advances in therapeutics, such as mycophenolate mofetil (MMF). To provide insight into rates of the clinical outcomes in current practice after biopsy-proven lupus nephritis, we used a prospective cohort of the patients with newly diagnosed lupus nephritis, treated with MMF and observed their outcomes.

Method

Twenty systemic lupus erythematosus (SLE) patients who began mycophenolate mofetil shortly after a biopsy-confirmed diagnosis of lupus nephritis were included in the analysis. There were five patients with class III, nine with class IV, four with class III-V, one with class IV-V and two with class V lupus nephritis. The initial dose of mycophenolate mofetil was 1000 mg twice daily. If no improvement was observed, the dose was increased to 1500 mg twice daily after one month. We estimated the survival function for the time until the urine protein/creatinine reached 0.50 grams or less, after starting MMF by using an approach that accommodated interval-censored data. We also evaluated the treatment response using five different sets of criteria for the response that have previously been used in the clinical trials. These included the Bristol Myers-Squibb (BMS), the American College of Rheumatology (ACR), the lupus nephritis assessment with rituximab (LUNAR ), the Aspreva Lupus Management Study (ALMS), and the Abatacept and Cyclophosphamide Combination Efficacy and Safety Study (ACCESS).

Result

We estimated that 52% of the SLE patients reached 0.50 grams of proteinuria within 51 days of starting mycophenolate mofetil (95% confidence interval 29%-74%) and 77% reached 0.50 grams or less within 260 days (95% confidence interval 57%-97%). The probability of response at 90 and 180 days was 5% and 33% (the Bristol Myers-Squibb), 26% and 57% (the American College of Rheumatology), and 11% and 28% (the lupus nephritis assessment with rituximab, the Aspreva Lupus Management Study and the Abatacept and Cyclophosphamide Combination Efficacy and Safety Study).

Conclusion

The complete renal response ranged from 28% to 57% at six months in the routine clinical practice, mirroring the results in randomized clinical trials. Regardless of the response measures, the complete renal response was slow and, by most indices, reached in only a minority of the patients by the end of six months of the induction therapy. This indicates the urgent need for the faster and more effective lupus nephritis treatments.

## Introduction

Lupus nephritis occurs in 50-60% of the systemic lupus erythematosus (SLE) patients within 10 years after the diagnosis [1-4]. The aggressive immunosuppressive therapy has improved the prognosis of the SLE patients with renal disease, but 5-20% still progress to end-stage renal disease within 10 years following the diagnosis of nephritis. The progression to renal failure in the patients with lupus nephritis is higher in the African-American patients [5]. There may also be a genetic basis for poor renal outcomes [6]. Tektonidou, et al. demonstrated a decreased risk of the end-stage renal disease (ESRD) between 1970 and 1990, followed by a plateau and then, possibly an increased risk in the recent years [7]. A socioeconomic gradient is apparent in the prevalence of lupus nephritis with increased prevalence in poorer geographic areas [8].

Proteinuria is a major predictor of the poorer renal outcomes and is a part of all outcome measures used in lupus nephritis clinic trials. The renal outcome data from the Euro-Lupus Nephritis Trial showed that the level of proteinuria at 12 months is the individual best predictor of long-term renal outcome in the patients with lupus nephritis [9]. Four recent lupus nephritis trials, however, have each used a different definition of complete renal response and none is equivalent to the published recommendations of the American College of Rheumatology [10].

In this report, we compared these renal outcome measures in the clinical practice to assess complete renal response after starting mycophenolate mofetil as initial therapy for class III, IV or V in the immunosuppressant naive patients with lupus nephritis. We used five outcome measures including the Bristol-Myers Squibb (BMS), the American College of Rheumatology (ACR ), the lupus nephritis assessment with rituximab (LUNAR ), the Aspreva Lupus Management Study (ALMS), and the Abatacept and Cyclophosphamide Combination Efficacy and Safety Study (ACCESS).

## Materials and methods

Patients included

The Hopkins Lupus Cohort is a prospective cohort of the patients with lupus who have been seen at the Johns Hopkins University since 1987 by one provider. The patients from the Hopkins Lupus Center were included in this analysis if they had biopsy-proven lupus nephritis class III-V, proteinuria of more than 0.50 grams at the time of the biopsy and were treated with mycophenolate mofetil (MMF) as their first immunosuppressive shortly after the biopsy. The patients were excluded if they were on hemodialysis, peritoneal dialysis, the patients who had a kidney transplant or had previous exposure to immunosuppressants for lupus nephritis. The 20 patients met the American College of Rheumatology and Systemic Lupus International Collaborating Clinics classification criteria for the SLE.

Clinical information collected

The baseline urine protein/creatinine was assessed prior to initiating mycophenolate mofetil and updated at the clinic visits, per protocol. The patients were scheduled to be seen monthly or quarterly depending on their clinical condition, however, the frequency of the actual visit varied.

Mycophenolate mofetil protocol

The initial dose of mycophenolate mofetil was 1000 mg twice daily. If the patient experienced no improvement, the dose was increased to 1500 mg twice daily after a month.

Treatment response definitions

Table 1 shows the criteria for the response based on previous studies. For the BMS outcome, the complete renal response required the urine protein/creatinine ratio to be ≤ 0.26 grams, and the estimated glomerular filtration rate (GFR) had to remain within 10% of the screening value. These criteria need to be achieved on two consecutive visits and no steroid taper was required. The LUNAR outcome required the serum creatinine level to be within 15% of the baseline level and the urine protein/creatinine ratio had to be ≤ 0.50 grams. The ACCESS complete response included the stabilization or improvement of the estimated glomerular filtration rate within 25% of the baseline value and the urine protein to creatinine ratio to be ≤ 0.50 grams. The urinalysis results were not included in the complete response criteria. The ALMS outcome included that the urine protein/creatinine ratio had to be ≤ 0.50 grams and also required normalization of the serum creatinine and urinalysis. The ACR outcome required that the urine creatinine ratio be ≤ 0.20 grams. The estimated glomerular filtration rate (eGFR) had to be within 25% of the baseline value, and normalization of urinalysis was required. The ACR outcome did not include steroid taper, but the LUNAR, ALMS, and the ACCESS outcome criteria all required that prednisone be successfully tapered to ≤ 10 mg/day.

**Table 1:** Definition of the complete response in lupus nephritis trials. BMS: the Bristol-Myers Squibb, ACR: the American College of Rheumatology, LUNAR: the lupus nephritis assessment with rituximab, ALMS: the Aspreva Lupus Management Study and ACCESS: the Abatacept and Cyclophosphamide Combination Efficacy and Safety Study (reprinted from the abatacept for the lupus nephritis 2012 [10], with permission from Dr. Wofsy, the author).

Criteria	Urine protein creatinine ratio	Creatinine or estimated glomerular filtration rate	Urinalysis, cells or casts	Steroid taper required	Criteria must be met on two successive visits
BMS	≤ 0.26	Within 10% of the screening or baseline value	Normal	No	Yes
ACR	≤ 0.20	Within 25% of the screening or baseline value	Normal	Not addressed	No
LUNAR	≤ 0.50	Within 15% of the screening or baseline value	Normal	Yes	No
ALMS	≤ 0.50	Normal	Normal	Yes	No
ACCESS	≤ 0.50	Normal or within 25% of the baseline value	Not a component	Yes	No

Statistical methods

The patients were seen at variable intervals, and if a patient satisfied the response criteria at a given visit, we assume that the response criteria were satisfied sometime between that visit and the previous visit (“interval censoring”). To estimate probabilities of the time, until a response taking into account interval censoring, we estimated the survival function by using the nonparametric method of Wellner and Zhan as implemented in the SAS (SAS Institute Inc, Cary, North Carolina) macro EMICM [11].

## Results

The 20 patients consisted of 18 females and were predominantly the African Americans (n=nine) and the Caucasian Americans (n=seven). The ages ranged from 18 to 70 years at the time of starting MMF. The probability of achieving a urine protein/creatinine ratio below 0.50 grams within 180 days was estimated to be 63% (95% confidence interval 41%-84%).

Table 2 shows the estimated probability of achieving a urine protein/creatinine ratio less than 0.50 grams within six months overall and in subgroups defined by the race and biopsy class. The estimated probability of achieving this goal was substantially lower among the African Americans.

**Table 2:** The estimated probability of achieving a urine protein/creatinine ratio below 0.5 within 180 days of the treatment initiation, overall and in subgroups.

Subgroup	Estimated probability (95% CI)
All patients (n=20)	63% (41%, 84%)
Race	
Caucasian American (n=7)	100%
African American (n=9)	40% (6%, 73%)
Class of lupus nephritis	
Class III (n=8)	72% (43%, 99%)
Class IV (n=10)	60% (31%, 89%)
Class V (n=6)	50% (10%, 90%)

Table 3 shows the estimated probability of achieving the response from each of the clinical trial sets. The probability of the response at 90 and 180 days was 5% and 33% (BMS), 26% and 57% (ACR), and 11% and 28% (LUNAR, ALMS, ACCESS). The estimate of long-term response was highest using the ACR renal response.

**Table 3:** The estimated proportion with remission by 90 and 180 days based on different definitions.

Response definition	Probability of the response within 90 days (95% CI)	Probability of the response within 180 days (95% CI)
BMS	5% (1%, 15%)	33% (9%, 37%)
ACR	26% (4%, 48%)	57% (35%, 79%)
LUNAR/ALMS/ACCESS	11% (1%, 25%)	28% (7%, 49%)

**Figure 1:**
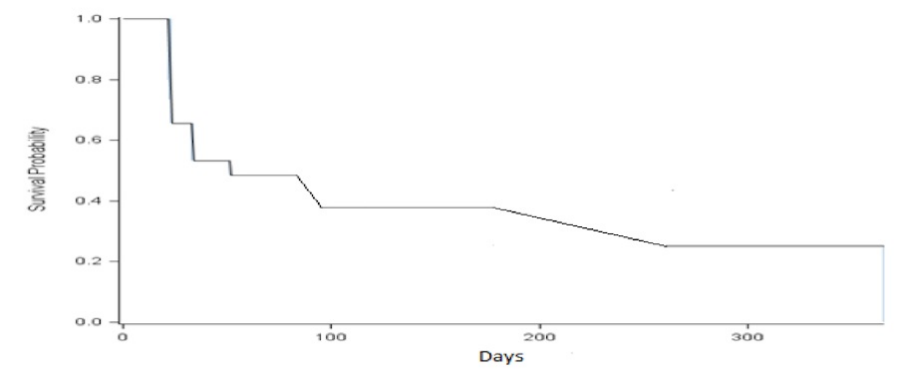
The timing (days) of the urinary protein to creatinine ratio reaches at or below 0.5 grams in the lupus nephritis.

Figure 1 shows the estimate of the probability that the patients would not achieve a urine protein/creatinine ratio below 0.50 grams at various time points after starting MMF. Fifty-two percent of the SLE patients reached 0.50 grams of proteinuria within 51 days of starting MMF (95% confidence interval 29%-74%). Seventy-seven percent reached 0.50 grams or less within 260 days (95% confidence interval 57%-97%). 

## Discussion

We report the outcomes of the treatment with mycophenolate mofetil as first-line therapy in Hopkins Cohort patients with class II, IV, or V lupus nephritis. Our study demonstrates that the majority of the patients in the clinical practice with newly diagnosed lupus nephritis (77%) can reach a urine protein to creatinine ratio of 0.50 grams or less within nine months after the initiation of mycophenolate mofetil. 

The risk of developing the end-stage renal disease is 2.6- to 5.6- fold greater in the African Americans than in the European Americans [6]. We also showed the response in the African American was slower compared to the Caucasian group. This response rate, however, appears to be lower in the African Americans.

The normalization of urine protein is clinically prognostic. An analysis of 90 patients participating in the Euro-Lupus Nephritis Trial revealed that a decrease in proteinuria of < 1 gm/day at six months predicted the long-term renal outcome for 10 years [12]. Dall’Era, et al. found that a rapid decline in the proteinuria within the first eight weeks of the treatment correlated strongly with achieving the response criteria at 24 weeks [13].

This is the first study to implement and compare the renal outcome measures developed for randomized clinical trials in routine clinical practice. Using response criteria from several recent clinical trials (BMS, ACR, LUNAR, ALMS, and ACCESS), we observed great variability in the response rates. The probability of response at 180 days was 33% (BMS), and 28% (LUNAR/ALMS/ACCESS). Interestingly, the probability of the response at 180 days was higher (57%) in the ACR response criteria, which could be due to the requirement in the reduction of urine protein creatinine ratio below 0.20 grams.

As we studied a small group of the patients, the validation of our findings should be considered using the long-term renal outcomes.

## Conclusions

The complete renal response with mycophenolate mofetil as the first-line therapy, in the routine clinical practice, was slow and by most indices, reached in only a minority of the patients by the end of six months of induction therapy. This indicates the urgent need for faster and more effective lupus nephritis treatments.
